# Diabetes and the diabetic foot in 2011

**DOI:** 10.1186/1757-1146-4-S1-A1

**Published:** 2011-05-20

**Authors:** Andrew JM Boulton

**Affiliations:** 1 University of Manchester, Manchester Royal Infirmary, Oxford Road, Manchester, M13 PWL, UK; 2 University of Miami, Miami, FL, USA

## 

We are facing a world epidemic of type 2 diabetes that is now one of the biggest threats to the health of the world for the 21^st^ Century. Previously published figures by the IDF are already gross underestimates of the current situation as recent studies from China suggest a prevalence of diabetes of almost 10%: thus the combined population of diabetic individuals from China and India is likely > 150 million individuals. In Australasia, diabetes prevalence is increasing, particularly amongst the native population. Diabetic foot problems have been recognised as increasingly important in recent years as they represent the commonest cause of hospital admission amongst diabetic patients in Western countries, are responsible for much morbidity and even mortality and are a major economic drain on the health care system. Data from the USA suggests > $30 billion is spent by the health care system on diabetic foot ulceration and amputation each year. In order to prevent foot ulcers there is a need to have a uniformly agreed screening programme that can be applied worldwide wherever the patient is screened, at home, in primary or secondary care. There is data to suggest that those patients with a history of previous neuropathic plantar ulcers who were randomised to self-monitor skin temperatures, were advised that if a difference of more than 1.5°C was found consistently between the two feet, they should rest: in that group compared to standard therapy, there was a highly significant reduction of recurrent ulcers from approximately 30% to 8% annual risk. There is also increasing evidence that the implementation of a multi-disciplinary foot care team across not only secondary but also primary care might help reduce the rate of amputations in people with diabetes. With respect to therapy, different modes of offloading remain the key to achieving appropriate healing in neuropathic ulcers. For complex diabetic foot wounds, negative pressure wound therapy using vacuum-assisted closure has supportive evidence from 2 randomised controlled trials in the area of wound healing. Most recently, hyperbaric oxygen therapy (HBO) whose efficacy has previously been questioned, has received support for improving wound healing in patients with ischaemic foot ulcers in whom vascular reconstruction is not possible. A well designed study from Sweden has confirmed the benefit of HBO and also suggests that this may result in few amputations and better quality of life. Other new areas such as gene therapy and other topical treatments are still awaiting confirmation of efficacy from properly designed randomised controlled trials rather than anecdotal case series.

